# Genetic analysis, structural modeling, and direct coupling analysis suggest a mechanism for phosphate signaling in *Escherichia coli*

**DOI:** 10.1186/1471-2156-16-S2-S2

**Published:** 2015-04-23

**Authors:** Stewart G Gardner, Justin B Miller, Tanner Dean, Tanner Robinson, McCall Erickson, Perry G Ridge, William R McCleary

**Affiliations:** 1 Microbiology and Molecular Biology Department, Brigham Young University, Provo, UT, USA; 2 Department of Biology, Brigham Young University, Provo, UT, USA

**Keywords:** PhoR, PhoU, PstSCAB, Pho regulon, two component signal transduction, PAS domain, direct coupling analysis

## Abstract

**Background:**

Proper phosphate signaling is essential for robust growth of *Escherichia coli *and many other bacteria. The phosphate signal is mediated by a classic two component signal system composed of PhoR and PhoB. The PhoR histidine kinase is responsible for phosphorylating/dephosphorylating the response regulator, PhoB, which controls the expression of genes that aid growth in low phosphate conditions. The mechanism by which PhoR receives a signal of environmental phosphate levels has remained elusive. A transporter complex composed of the PstS, PstC, PstA, and PstB proteins as well as a negative regulator, PhoU, have been implicated in signaling environmental phosphate to PhoR.

**Results:**

This work confirms that PhoU and the PstSCAB complex are necessary for proper signaling of high environmental phosphate. Also, we identify residues important in PhoU/PhoR interaction with genetic analysis. Using protein modeling and docking methods, we show an interaction model that points to a potential mechanism for PhoU mediated signaling to PhoR to modify its activity. This model is tested with direct coupling analysis.

**Conclusions:**

These bioinformatics tools, in combination with genetic and biochemical analysis, help to identify and test a model for phosphate signaling and may be applicable to several other systems.

## Background

Adapting to changes in the environment is one of the hallmarks of life. For all life, phosphate is an essential nutrient. Bacteria have several mechanisms to scavenge phosphate that are only expressed when the level of available environmental phosphate is limited: including a phosphate specific ABC transporter complex (PstSCAB) and a periplasmic phosphate scavenging enzyme (alkaline phosphatase (AP); the product of the *phoA *gene) [1]. Expression control of these genes is essential for optimal growth and has been implicated in the regulation of pathogenesis in several organisms [2,3].

In *Escherichia coli*, a classic two-component signal transduction system, composed of the PhoR histidine kinase and the PhoB response regulator, is responsible for expression control of a group of genes called the Pho regulon. PhoR consists of an N-terminal membrane-spanning region, as well as cytoplasmic PAS, DHp and CA domains. The PAS domain was named for the *Drosophila ***P**er, **A**rnt, and **S**im proteins, in which this domain was originally described and has been found in many signaling proteins. Many PAS domains bind cofactors such as heme [4]. The DHp domain is conserved in histidine kinases and functions in **d**imerization and contains the site of **h**istidine **p**hosphorylation. The CA domain is the **c**atalytic, **A**TP-binding part of the protein. PhoB consists of an N-terminal phosphorylation domain that receives a phosphoryl group from PhoR and a C-terminal DNA binding domain. In low phosphate conditions, PhoR acts as a PhoB kinase. Upon phosphorylation, PhoB recruits RNA polymerase to promoters of the Pho regulon that contain a Pho box. In high phosphate conditions, PhoR acts as a phospho-PhoB phosphatase and removes the phosphate from PhoB to keep the expression level of Pho regulon genes very low. One unanswered question with this system is how PhoR perceives external phosphate concentrations. PhoR lacks a significant periplasmic domain that could detect phosphate abundance outside the cell. Past work has shown that the PstSCAB transporter and the PhoU protein play important roles in phosphate signaling to PhoR [1]. The mechanism of this signal has not been fully elucidated.

A deletion mutation of the *phoU *gene leads to poor growth and the frequent development of compensatory mutations in the other Pho regulon expression control genes, *pstSCAB*, *phoR*, and *phoB *[5]. The poor growth phenotype is likely due to overexpression and under-regulation of a functional PstSCAB transporter [6], which leads to phosphate poisoning when cells are grown in high phosphate environments [7]. Reference [7] proposed that PhoU modulates phosphate transport through the PstSCAB complex by inhibiting transport when internal phosphate levels are too high.

**Figure 1:**
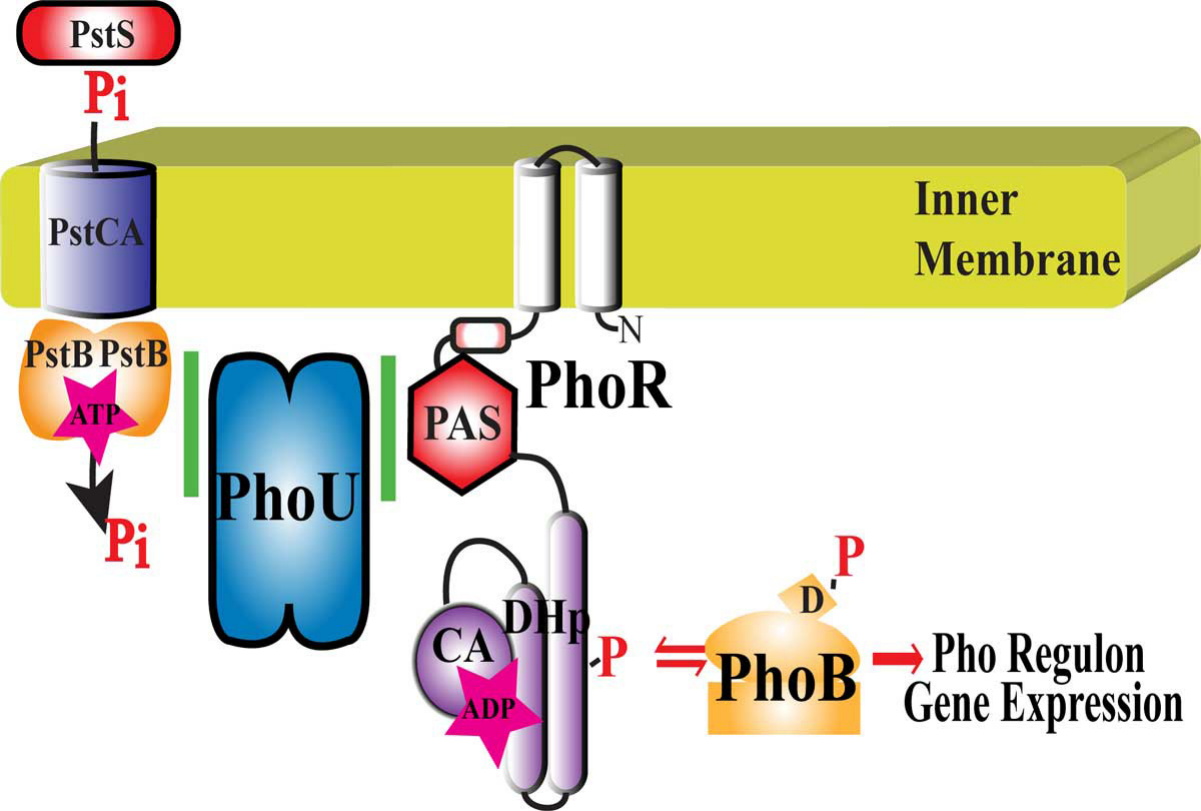
**Pho regulon expression control**. Proper control of Pho regulon expression involves the histidine kinase, PhoR that has a Per-Arnt-Sim (PAS), dimerization/histidine phosphorylation (DHp), and catalytic (CA) domains; the response regulator PhoB (with a conserved aspartate (D) residue that is phosphorylated); the phosphate specific ABC transporter composed of PstS (Periplasmic phosphate binding protein), PstC and PstA (proteins that form the pore in the inner membrane), and PstB (the ATP binding portion of the transporter); and PhoU (a negative regulator of PhoR).

Recently, PhoU was shown to directly interact with PstB and PhoR [8]. This observation suggested a model that PhoU interacts with PstB to sense environmental phosphate levels and that it passes that signal along to PhoR to modulate its kinase/phosphatase activities (Figure 1). To further characterize these interactions, this work analyzes several *phoU *mutants for signaling activity, interactions with PhoR, and interactions with PstB. A scanning mutagenesis screen of the PAS domain of PhoR identified potential residues important for interaction with PhoU. We modeled potential docking interactions between PhoR and PhoU. One of the docking models showed a close proximity of identified residues in PhoU with a predicted interaction loop of the PAS domain of PhoR. To validate this model, we performed a Direct Coupling Analysis (DCA) between PhoR and PhoU using sequences from the gammaproteobacteria. The DCA results are consistent with the proposed docking model and may point to a mechanism of action for PhoU in controlling the opposing kinase and phosphatase activities of PhoR.

## Methods

### Strains, plasmids, and reconstructing of signaling system

Plasmids that were used include pKG116[9], p116U2[7] (a pKG116 with the *phoU *gene under salicylate expression control), p116A147E, p116U2 A147K, p116U2 R148A, p116U2 R148E, p116U2 D150A (all the mutant p116U2 plasmids were constructed with QuikChange site-directed mutagenesis kit from Agilent Technologies and verified by DNA sequence analysis as described in [8] (primers listed in Table S1 found in additional file 1), pRR48[10], and p48SCAB (a pRR48 with the *pstSCAB *genes inserted also as described in [8] (primers are listed in Table S1 found in additional file 1). For signaling analysis, combinations of p48SCAB and a pKG116 derived plasmid were introduced into the *E. coli *strain BW26337 (having a chromosomal deletion of *pstSCABphoU *constructed from the wildtype strain BW25113 [11]). The cultures were grown at 37°C with shaking overnight in morpholinepropanesulfonic acid (MOPS) defined medium with 0.06% glucose and 2.0 mM phosphate (a high phosphate minimal media) [8].

### Growth of PhoU A147E mutant

STAC and STACΔphoU [8] were used with pKG116, p116U2, or p116U2 A147E (a p116U2 plasmid mutated with QuikChange site-directed mutagenesis kit from Agilent Technologies and verified by DNA sequence analysis (primers listed in Table S1 found in additional file 1)). Triplicate overnight cultures were grown in LB media with 100 µM IPTG and the OD_600 _of a 1:5 dilution of culture was measured. Values were adjusted for dilution and averages are reported with error bars representing the standard deviation.

### Alkaline phosphatase assays

We based our Alkaline Phosphatase (AP) Assays on a 96 well plate β-Galactosidase Activity assay previously described [8]. Cultures were grown overnight at 37°C with shaking. The OD_600 _was read on a 1:4 dilution of the culture. 1 ml of culture was collected and resuspended in 900 µl of 1 M Tris-HCl pH 8.2. 1 drop of 0.1% sodium dodecyl sulfate and 2 drops of chloroform were added to each tube and tubes were vortexed vigorously for 15 sec. Tubes were then spun for 1 min at 16,000 × g and 200 µl of each sample was loaded into a well of a 96 well flat bottomed plate. 40 µl of 20 mM p-Nitrophenyl phosphate in 1 M Tris-HCl pH 8.2 was added. The OD_420 _values were read once a minute for 20 min at 37°C. Units of activity were calculated as (1000 × slope of a line fit to OD_420 _in mOD/min)/(4 × OD_600 _of 1:4 dilution of the overnight culture). Cultures were grown in triplicate and the average is reported with error bars representing the standard deviation.

### BACTH and β-galactosidase assays for scanning mutagenesis

BACTH analysis and β-Galactosidase Assays were performed as described in [8]. Briefly, using the QuikChange Lightning Site-Directed Mutagenesis Kit from Agilent Technologies, the p18CRN-PAS plasmid was mutated to change every two amino acids of the PhoR PAS domain to code for alanine-cysteine (primers listed in Table S1 found in additional file 1). For example, p18CRN-PAS 109 had PhoR D109A and A110C changes. These residues were chosen because they are not predicted to cause major secondary structural changes. Each p18CRN-PAS plasmid of the library of alanine-cysteine mutants was transformed with pKT25phoU into the tester strain, BTH101 from EuroMedex. Cultures were grown in triplicate in LB and assayed as described in [8]. The percent of PhoU/PhoR Interaction was found by dividing the average activity of each sample with the activity of an unmutated p18CRN-PAS control and multiplied by 100.

### Protein structure modeling and protein docking modeling

We used the ClusPro webserver [12-15] to dock structures of a dimer of the cytoplasmic portion from *E. coli *PhoR (structure modeled from the structure for VicK from *Streptococcus mutans *[16]), and PhoU (modeled from *Thermatoga maratima *PhoU [17]) using **P**rotein **H**omology/analog**Y R**ecognition **E**ngine V 2.0 (Phyre2) [18].

### Direct coupling analysis

We used PhoU and PhoR sequences from 196 species of bacteria from the gammaproteobacteria group. Sequences for proteins were collected using the Kegg webserver (http://www.kegg.jp/). We used a list of PhoR and PhoB orthologs to identify species where both annotated PhoR PhoB encoding genes were found on the chromosome. The PhoR and PhoU sequences were collected manually using the predicated protein domains and the genomic context of the gene to select genes that were most likely correctly annotated. For example, for this analysis we were not interested in histidine kinases annotated as PhoR from species that did not have a *phoU *gene on the chromosome. Sequences were concatenated starting with PhoU, followed by PhoR from the same species. Twenty alanine residues were artificially placed between the two protein sequences to aid in differentiating the PhoU from the PhoR after the alignment. Using MAFFT version 7 (**website is **http://mafft.cbrc.jp/alignment/server/), we were able to create an alignment file to use in DCA analysis. A MATLAB implementation of direct coupling analysis reported in [19] distinguished differences between the direct information and mutual information between all protein residues. The command line argument used was matlab-nodisplay-nojvm-nosplash-r "dca $INPUT_ALIGNED_FASTA $OUT_FILE". The DCA implementation used the input aligned fasta file in its calculations, outputting a file with N(N-1)/2 (N = length of the sequences) rows and four columns: residue i (column 1), residue j (column 2), MI(i,j) (Mutual Information between i and j), and DI(i,j) (Direct Information between i and j). All inserts columns were removed from the alignment during the analysis. Concluding this analysis, a simple python script was used to remove all rows in which residues i and j were within five residue numbers of each other. This was done to eliminate false positive residue interactions caused by their proximity to other amino acids. Mutual information was later screened to signify relatedness between two residues.

## Results and discussion

**Figure 2:**
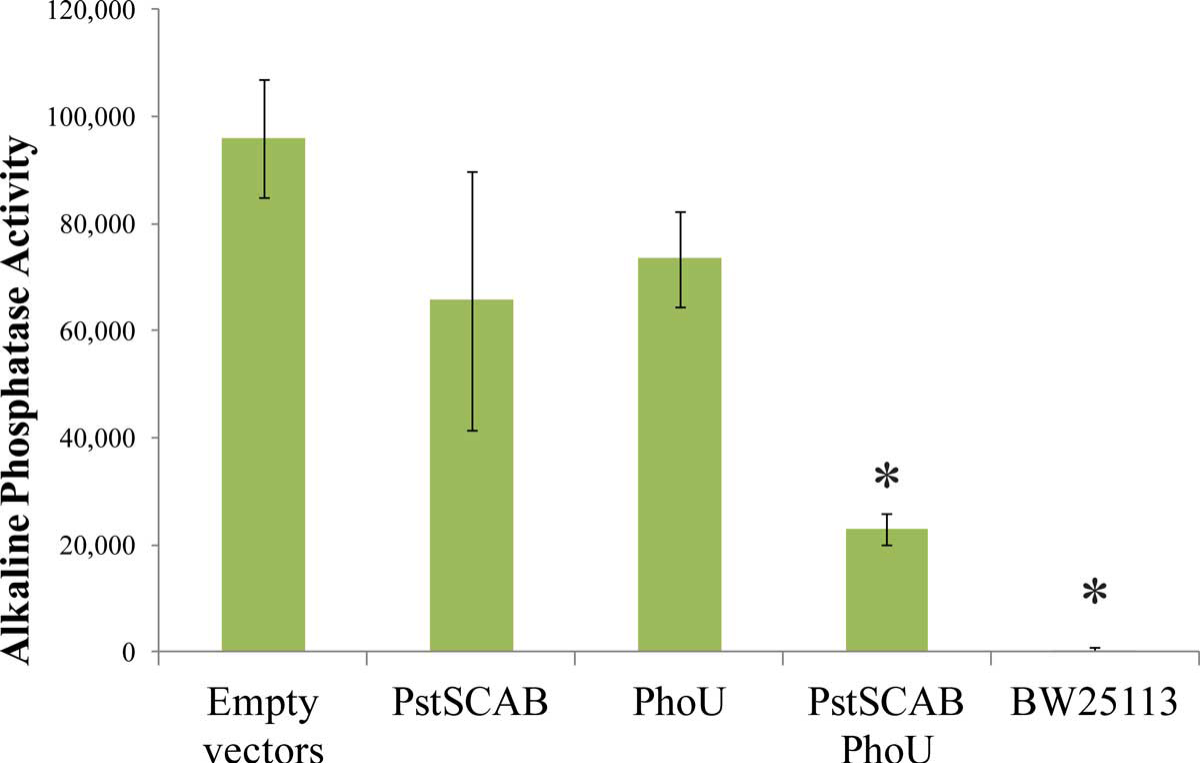
**Signaling necessity and sufficiency of PhoU and PstSCAB**. Triplicate cultures of BW26337 (a Δ*pstSCABphoU *strain) cells with pKG116 and pRR48 (Empty vectors), pKG116 and pRR48SCAB (PstSCAB), p116U2 and pRR48 (PhoU), p116U2 and pRR48SCAB (PstSCAB PhoU) and BW25113 (a wild-type strain) were grown in LB and Bacterial Alkaline Phosphatase activity was assayed. Error bars represent the standard deviation. * = p-value of < 0.05 compared to the Empty vectors with a two-tailed T-test.

Mutations in any of the *pstSCAB *transporter genes or *phoU *lead to loss of phosphate signaling. To determine whether PhoU could act independently of the PstSCAB transporter, we expressed these two genetic entities from separate plasmids in *E. coli *strain BW26337, which contains a deletion of the *pstSCABphoU *operon and tested strains for control of the Pho regulon. We cloned the *pstSCAB *genes into the pRR48 plasmid (Amp^R^) [10] and the *phoU *gene into the compatible pKG116 plasmid (Cam^R^) [9]. Control of the Pho regulon was analyzed by determining Alkaline Phosphatase (AP) expression. Figure 2 shows that AP expression was unregulated when cells were grown under high phosphate conditions in strains expressing neither protein (containing two empty vectors), or expressing each protein individually. However, when both proteins were expressed in the same cell, AP expression levels were significantly reduced. These results show that *phoU *expression is necessary but not sufficient for proper phosphate signaling and are consistent with a model that these proteins act together in signal transduction.

Early studies of phosphate homeostasis in *E. coli *isolated mutants that constitutively expressed AP [20]. One mutant, named C4 [21] was characterized [22] and was later named *phoU35 *because it was independent of the phosphate transport genes [23]. When the genes of the *pstSCABphoU *operon were sequenced and the *phoU35 *mutant was analyzed, they found this mutation encoded a change from alanine at position 147 to glutamic acid (A147E) [24]. Since a *phoU *deletion mutation results in loss of phosphate signaling and causes a severe growth phenotype, but the *phoU*35 allele was only reported to cause a loss of signaling without the accompanying poor growth, we hypothesized that the *phoU*35 mutation may disrupt PhoU's interaction with PhoR, preventing the signal for the switch to PhoR phosphatase activity, but may maintain the interaction with PstB, limiting excess transport of phosphate into the cell during phosphate replete conditions.

**Figure 3:**
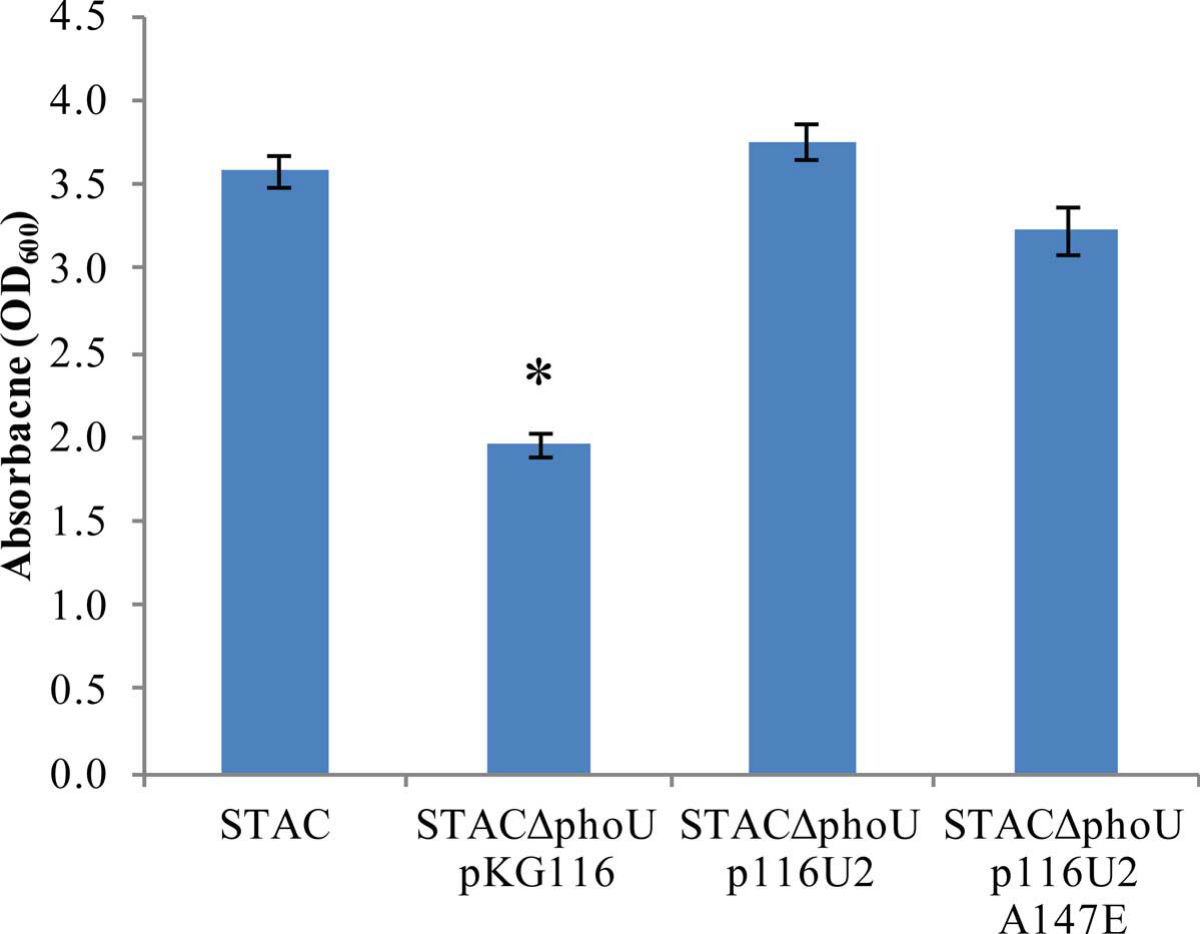
**Growth yield of overnight cultures**. Triplicate cultures were grown overnight in LB and the absorbance of 600nm wavelength of light for each culture was recorded. Error bars represent standard deviations. * = p-value of < 0.01 compared to STAC with a two-tailed T-test.

We wanted to confirm that strains expressing the *phoU*35 allele do not show a severe growth phenotype. We employed a *phoU *deletion strain in which the normal *pstS *promoter was replaced by the *tac *promoter to uncouple expression of this operon from mutations in any of its genes (STACΔphoU) [8]. By growing this strain in the absence of IPTG the accumulation of compensatory mutations was avoided. Figure 3 shows the results of characterizing the growth yield of various cultures grown in a high-phosphate medium with IPTG overnight with shaking. The STAC strain with a wildtype copy of *phoU *still in the chromosome grew normally. We observed a significant growth defect when the *phoU *knockout strain containing an empty vector, pKG116, was grown in identical conditions. When the p116U2 plasmid was introduced into this strain, expressing wild-type *phoU*, and grown under identical conditions, no growth defect was observed. Moreover, when the *phoUA147E *allele was introduced into the strain, the growth phenotype was similar to that of wild-type *phoU*. These results confirm the lack of growth defect in the *phoU35 *mutant and are consistent with the hypothesis that the A147 residue of PhoU is important for interactions with PhoR.

**Figure 4:**
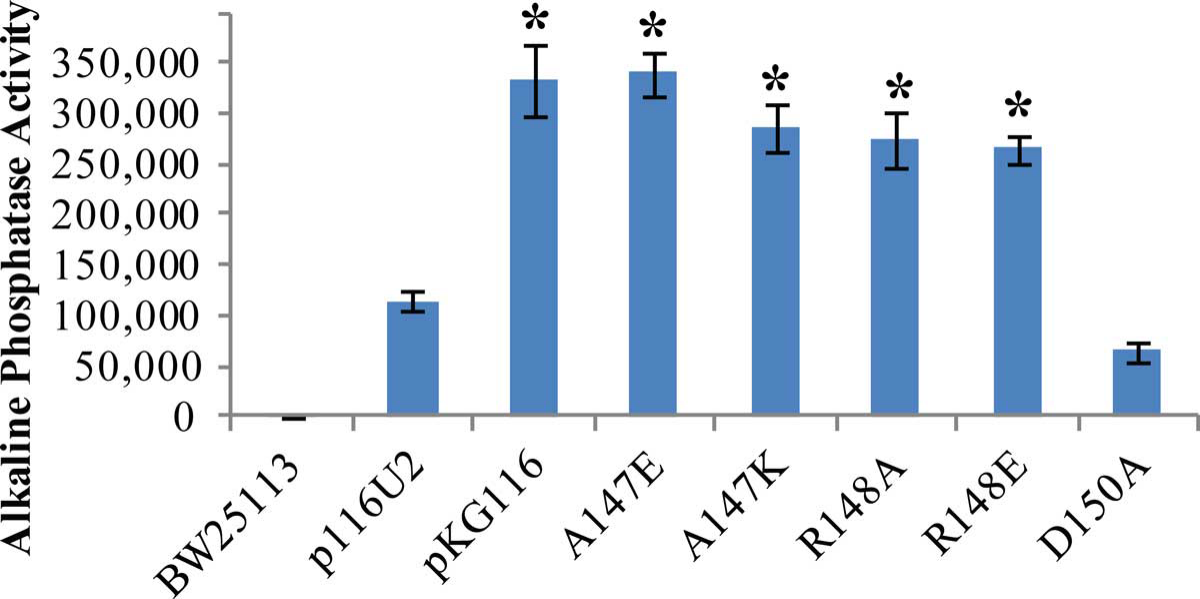
**Signaling activity of various PhoU mutants**. Triplicate cultures were grown in LB and assayed for Bacterial Alkaline Phosphatase activity. BW25113 is a wildtype control. Other strains were STACΔphoU with p116U2 (wildtype *phoU*), pKG116 (empty parent plasmid), and various p116U2 plasmids with specific mutations in *phoU *(A147E, A147K, R148A, R148E, and D150A). Error bars represent standard deviations. Values significantly greater than p116U2 are labeled with * = p-value of < 0.001 compared to p116U2 with a two-tailed T-test.

To verify that the *phoU*35 allele caused a signaling defect when expressed from a multicopy plasmid, we tested AP expression in the strains constructed for the previous experiments. Figure 4 shows that high AP levels were observed in the STACΔphoU pKG116 strain and the STACΔphoU p116U2 A47E strain grown under high phosphate conditions, but that reduced AP levels were observed in the STACΔphoU p116U2 strain. This showed that PhoUA147E could not regulate PhoR and was consistent with the model presented. We wondered if other mutations that caused changes in the *phoU *protein in the vicinity of A147 would also display a signaling defect. We therefore introduced additional mutations by site-directed mutagenesis in the region near A147 (A147, R148, and D150) to create versions of PhoU with different charges and sizes of amino acid side chains in this region. The mutations that we created were A147K, R148E, R148A, and D150A. The rightmost four bars in Figure 4 show that the A147K, R148E and R148A mutants lost phosphate signaling activity as they expressed elevated AP levels, but that the D150A mutation retained signaling activity. These results suggest that both A147 and R148 are important for interactions with PhoR.

The combination of no growth inhibition and disrupted signaling are the expected phenotypes of a *phoU *mutant that can interact with PstB to limit phosphate transport but can no longer interact with PhoR to signal the phosphate level.

**Figure 5:**
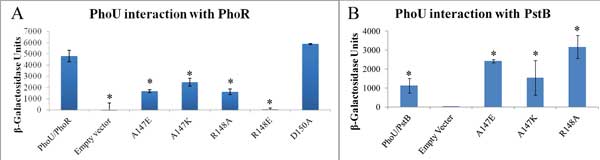
**PhoU mutant interactions with PhoR and PstB**. Values shown are the average of triplicate samples with the error bars representing standard deviation. A. BACTH analysis of PhoU/PhoR interaction. Various combinations of PhoU and mutants or an empty vector negative control were tested for interaction with PhoR. Activity of β-Galactosidase correlates with protein/protein interaction. * = p-value < 0.01 compared to PhoU/PhoR with a two-tailed T-test. B. This chart shows the interaction of PstB with various PhoU mutants and an empty vector negative control. * = p < 0.05 compared to Empty Vector with a two-tailed T-test.

The combination of no growth inhibition and disrupted signaling are the expected phenotypes of a *phoU *mutant that can interact with PstB to limit phosphate transport but can no longer interact with PhoR to signal the phosphate level. We further examined this hypothesis by using a bacterial adenylate cyclase two-hybrid (BACTH) system to test for interactions between mutant versions of PhoU and PstB and PhoR (Figure 5A and 5B).

The BACTH system employs the separable T18 and T25 domains of adenylate cyclase from *Bordetella pertussis*. When these two domains are in close proximity they create an active enzyme that produces cAMP. By creating gene fusions in which protein domains are connected to the T18 and T25 fragments, cAMP production is a measure of whether the fused proteins interact. Since cAMP binds to the CRP protein, cAMP can be measured indirectly by assaying β-galactosidase. We previously used this method to show interactions between the wild-type version of PhoU and PstB and various domains of PhoR [8].

Figure 5A shows the results of β-galactosidase activity assays of various BACTH strains. Each sample is BTH101 strain containing one plasmid that expresses the T18 domain fused to PhoR and another plasmid that expresses either the T25 domain alone (Empty vector), T25 fused to PhoU (PhoU/PhoR), or various PhoU mutant proteins (A147E, A147K, A148A, R148E, and D150A). The A147 and R148 mutants of *phoU *had a significantly weaker interaction with PhoR (as represented by low β-galactosidase activities) and the D150A mutant retained PhoR interaction. We also tested these plasmids expressing the T25-PhoU fusions for interaction with a T18-PstB fusion protein. Interestingly, the A147 and R148 mutant proteins maintained interactions with PstB (Figure 5B). This implies that the loss of interaction with PhoR is not due to protein instability or radical protein misfolding.

**Figure 6:**
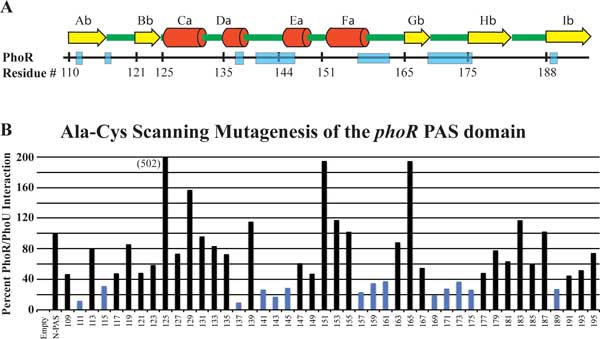
**PAS domain scanning mutagenesis**. A. Predicted secondary structure of the PhoR PAS domain with alpha helixes labeled with yellow arrows and β-sheets labeled with red cylinders. B. Scanning mutagenesis of the PhoR PAS domain used BACTH to identify regions of the protein that are essential for interaction with PhoU. Every two amino acids were changed to code for alanine and cysteine. Each construct was tested in triplicate for interaction with PhoU. Blue bars represent samples that had less than 40% of the activity of unmutated PhoR.

Previous work showed that the PhoR/PhoU interaction was dependent on the PAS domain of PhoR [8]. We used scanning mutagenesis of a plasmid expressing a T18-PhoR N-PAS (the portion of the PhoR protein from the C terminus through the PAS domain, but without the CA and DHp domains) domain fusion protein to identify the residues within this domain that are important for the interaction. Every two amino acids of the PhoR PAS domain were mutated to alanine and cysteine residues by sequentially replacing the six bases encoding adjacent codons with a *SphI *restriction site. The mutant versions were then introduced into the appropriate tester strain expressing wild-type *phoU *fused to the T25 fragment of adenylate cyclase and β-galactosidase assays were performed. Many mutants lost the ability to interact with PhoU as indicated by significantly reduced β-galactosidase levels (shown as blue bars in Figure 6B). Among the mutations there were several regions where neighbouring residues showed loss of interaction (for example, residues 141-146, 157-162 and 169-176). There were also several single mutations (residues 111, 115, 189) that reduced protein interactions.

**Figure 7:**
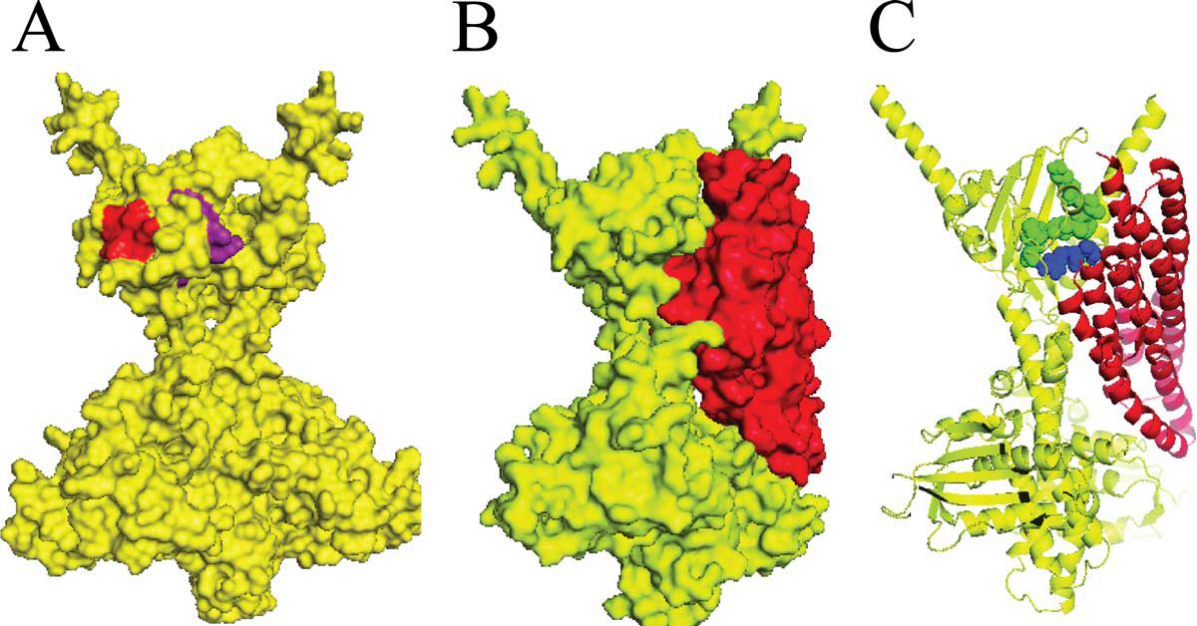
**Structures of modeled PhoR and PhoU**. A. Interaction sites identified from scanning mutagenesis that are found on the surface of a modeled PhoR structure, 141-146 are red and the 157-162 residues are purple. B. Shows the protein surface predictions of the PhoU/PhoR docking model. PhoR is colored yellow and PhoU is colored red. C. A cartoon of protein backbones with PhoU A147 and R148 shown as spheres in blue and PhoR K157-S162 shown as green spheres.

We modeled the cytoplasmic portion of the *E. coli *PhoR structure based upon the structure of the VicK protein from *Streptococcus mutans *using the Phyre2 webserver (Figure 7A). This modeled structure predicts that the 141-146 and the 157-162 regions of PhoR have residues that are exposed to the surface and face outwards, consistent with an interaction with another protein (Figure 7A, the residues colored red and purple respectively.) The 111, 115, 189, and the 165-176 mutations all map to regions of PhoR that are predicted to lie along a β-sheet that has been shown in other histidine kinases to be important for PAS domain dimerization contacts. For this reason, these mutations may not be involved in PhoU/PhoR interactions, but for maintaining a proper protein conformation.

We docked a modeled *E. coli *PhoU protein onto the modeled PhoR dimer. The server returned ten potential PhoR/PhoU models with most of the models being unreasonable because they docked PhoU onto the part of PhoR that interacts with the cytoplasmic membrane. One of the reasonable models showed an interesting interaction between PhoR and PhoU (Figure 7B). In this model the PhoU residues that were identified through genetic analysis as important for interactions with PhoR were in close proximity to regions of the PhoR PAS domain that were identified by scanning mutagenesis. Figure 7C shows that some of the PhoR 157-162 residues (shown in green) appear to interact with the A147 and R148 residues of PhoU (shown in blue).

Support for this interaction model was obtained through a bioinformatic method, called direct coupling analysis (DCA) that identifies covariance between residues. DCA identifies which residues from a sequence tend to co-evolve with any other residue by measuring the predictive power of one residue on another. This can identify direct interactions between residues, such as residues that are directly involved in protein/protein interactions. Also, residues that are involved in indirect interaction (for example residues that play a role in proper structural conformation) and mechanistic interactions (as would be found in residues associated with an enzyme active site) are identified with DCA [25]. Mutual information (MI), which identifies direct and indirect coupling of residue pairs, has been used to identify both intradomain and interdomain interactions [26-32]. Direct information (DI) is a value that attempts to eliminate the indirect coupling of MI due to neighbouring residues. Biologically, MI may identify residues that play a role in interaction through indirect means that may not be identified with DI. We used MI to sort our DCA results to identify the residues that play an important role in PhoU/PhoR interaction,

We analyzed sequences of PhoU and PhoR from 196 gammaproteobacteria. We concatenated the *phoU *and *phoR *sequences from each species with 20 ala residues in between the two genes to aid in alignment. We aligned the sequences and then compared all the residues with DCA.

**Figure 8:**
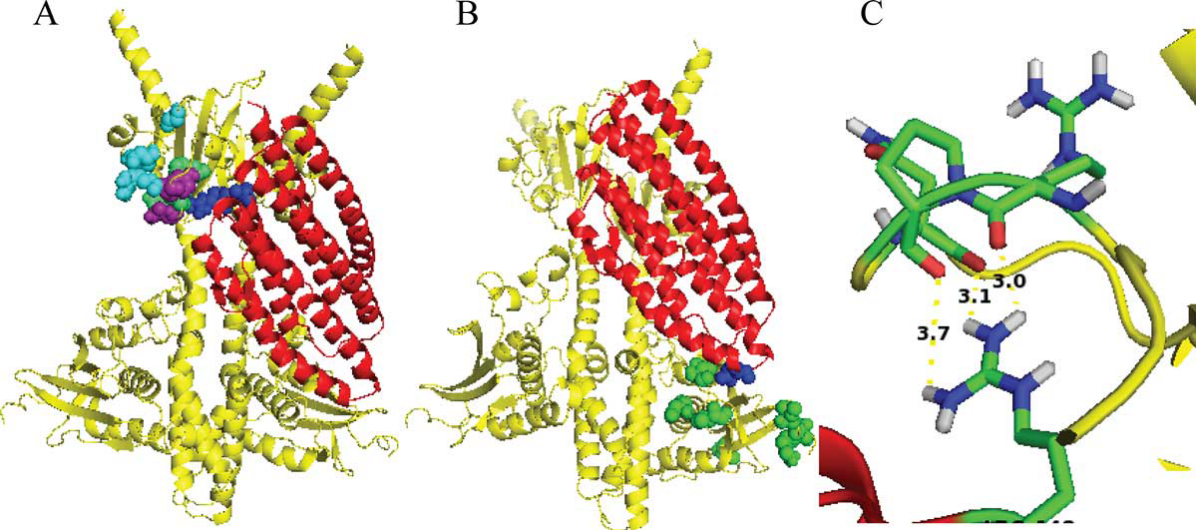
**Residues identified from DCA**. A. PhoR in yellow and PhoU in red with A147 and R148 highlighted in blue and the top five DCA residues of PhoR PAS domain highlighted as spheres (residues that co-vary with A147 are green, R148 are cyan, and residues that co-vary with both are purple). B. PhoU E121 highlighted in blue and the top seven residues in the DCA analysis of the PhoR CA domain highlighted in green spheres. C. PhoU R148 potential interactions with the side chain of PhoR PAS R163, P164, and N166 residues (distances labeled are Å).

Our previous work identified PhoU A147 and R148 residues as being involved in interaction with PhoR. To isolate residues from the PhoR PAS domain that show covariance with PhoU A147 and R148, we sorted the top co-varying residues of the PhoR PAS domain by MI for both A147 and R148 positions of PhoU. The top ten hits are shown in Table 1 and Table 2. Several of these co-varying residues fall in the same region of the PAS domain of PhoR identified by the BACTH analysis. When the top five residues for A147 and R148 are highlighted in the docking model, we see that many cluster near the predicted interaction surface. Given that some of the residues for A147 overlap with the R148 residues we colored residues that co-vary with A147 green, residues that co-vary with R148 cyan, and residues that co-vary with both purple (Figure 8A).

**Table 1:** DCA of PhoU A147 vs.PhoR PAS domain.

PhoR	PhoU	Mutual information	Direct Information	Distance (Å)
Y149	A147	0.5876	0.0016	12.1
N166	A147	0.5475	0.0006	14.7
F161	A147	0.5189	0.0005	12.2
P164	A147	0.5045	0.0009	12.9
R163	A147	0.4823	0.0005	12.1
G118	A147	0.4796	0.0011	19.0
Q186	A147	0.4565	0.0009	16.6
L187	A147	0.4459	0.0012	13.7
D160	A147	0.4442	0.0006	6.0
Q154	A147	0.4438	0.0008	10.9

**Table 2:** DCA of PhoU R148 vs.PhoR PAS domain.

PhoR	PhoU	Mutual information	Direct Information	Distance (Å)
R148	R148	0.4980	0.0045	11.4
Y149	R148	0.4927	0.0006	7.2
G118	R148	0.4121	0.0011	16.3
N145	R148	0.4082	0.0011	12.9
N166	R148	0.3901	0.0007	3.1
P164	R148	0.3861	0.0004	2.0
Q154	R148	0.3831	0.0006	12.1
N139	R148	0.3795	0.0004	21.9
P150	R148	0.3781	0.0017	12.0
R163	R148	0.3767	0.0003	3.0

With the docking model it appears that the loops on the opposite side of PhoU from the PAS interacting surface are in close proximity to the CA domain of the opposite PhoR of the dimer. To observe any covariance between PhoU and PhoR at these surfaces, we compared the PhoU E121 residue to the CA domain with DCA. We see that the top PhoR CA domain residues (shown as green spheres) that co-vary with E121(shown as blue spheres) cluster at the surface of PhoR nearest to PhoU E121 (Table 3 Figure 8B).

**Table 3:** DCA of PhoU E121 vs.PhoR CA domain

PhoR	PhoU	Mutual information	Direct Information	Distance (Å)
K428	E121	0.512	0.003	17.8
N429	E121	0.489	0.001	19.2
D316	E121	0.475	0.001	10.6
T340	E121	0.461	0.004	23.8
I426	E121	0.460	0.003	10.2
L274	E121	0.453	0.001	6.4
T271	E121	0.453	0.001	3.4
E375	E121	0.451	0.003	22.3
S430	E121	0.443	0.001	20.1
K277	E121	0.437	0.001	14.3

For a more complete analysis, we also sorted the potential interacting pairs by DI and found that several of the same residues were found in the top pairs (Table 4 and Table 5). In the tables, residues that were also found in the top ten residues of MI sorted data are bolded. Several other residues sorted by MI show relatively large DI values (Table 1 and Table 2). Also, it is interesting that many of the interacting pairs sorted by DI fall into the loop regions identified by the PAS domain scanning mutagenesis (Figure 6).

**Table 4:** DCA of PhoU A147 sorted by Direct Information

PhoR	PhoU	Mutual information	Direct Information	Distance (Å)
R148	A147	0.4267	0.0028	16.9
T195	A147	0.1738	0.0019	18.6
**Y149**	A147	0.5876	0.0016	9.3
L133	A147	0.2515	0.0016	20.9
I120	A147	0.1997	0.0015	20.0
E151	A147	0.4273	0.0014	8.0
L146	A147	0.3957	0.0014	15.8
L126	A147	0.2256	0.0014	22.2
V168	A147	0.3870	0.0014	15.0
F121	A147	0.2997	0.0013	22.1

Bolded PhoR residues are also in the top ten residues when sorted by Mutual Information

**Table 5:** DCA of PhoU R148 sorted by Direct Information

PhoR	PhoU	Mutual information	Direct Information	Distance (Å)
**R148**	R148	0.4980	0.0045	11.4
I143	R148	0.2236	0.0019	11.2
L167	R148	0.2752	0.0017	5.4
**P150**	R148	0.3781	0.0017	11.6
L126	R148	0.1135	0.0016	14.5
E176	R148	0.3762	0.0014	13.5
I120	R148	0.2126	0.0013	15.2
F152	R148	0.2972	0.0012	13.4
**N145**	R148	0.4082	0.0011	12.9
**G118**	R148	0.4121	0.0011	16.3

Bolded PhoR residues are also in the top ten residues when sorted by Mutual Information

We noticed that there are few residues that appear to interact based on the docking model and DCA that did not show loss of function in our scanning mutagenesis (~PhoR163-166). To better understand these results we looked closer at the residues in our docking model structures. When we highlight the side chain of the PhoU R148 residue and the side chains of residues in positions 163, 164, and 166 of the PhoR PAS domain, it appears that the R148 may interact with the amino acid backbone for these sites and not the side chain residues as they appear to point away from the PhoU structure (Figure 8C). Changing the side chains of these amino acids in our scanning mutagenesis of the PAS domain may not disrupt PhoU/PhoR interactions, which explains our scanning mutagenesis results.

Additionally, when distances between predicted interacting pairs is measured, several residues are found near R148 (within ~8Å) but few are found that near to A147. One explanation for this is that PhoU A147 itself does not directly interact with PhoR, but mutations of PhoU A147 may affect the ability of R148 to interact with PhoR. For example, the A147E mutation places a large acidic side chain right next to the R148 basic side chain and may disrupt proper PhoU/PhoR interaction and phosphate signaling. Using MI to sort our DCA results allows for identifying residues like this that are important for proper interaction but may not be directly involved. This phenomenon may also explain some of the PhoR PAS residues identified with DCA, like PhoR G118. The PhoR G118 residue is not found very near to PhoU R148 or A147 in our docking model (Table 1 and Table 2). However, G118 is on the surface of the PhoR PAS domain on a loop between α-helices that appears to form the top of the PhoU binding pocket. One would expect that mutations of a highly flexible glycine at this position could be associated with compensating mutations of PhoU in the R148 region.

## Conclusions

Our results confirm that PhoU is necessary for proper signaling in high phosphate, but that the PstSCAB transporter is also necessary. We have identified the A148 and R149 residues of PhoU as being important for interaction with PhoR. Using scanning mutagenesis, we identified residues of the PhoR PAS domain essential for interaction with PhoU. A docking model was identified and tested with DCA. The docking model shows PhoU interacting with both the PAS domain and the CA domain of PhoR, pointing to a potential mechanism for PhoU mediated switching of PhoR's kinase to phosphatase activity.

The switch from kinase to phosphatase activity for histidine kinases is dependent upon the conformation between the CA and DHp domains [33,34]. The CA domain must remain flexible for proper kinase function. Also, the DHp domain was previously shown biochemically to be the portion of PhoR that has phospho-PhoB phosphatase activity [35]. It is possible that when PhoU interacts with PhoR, that interaction may constrain the CA domain to inhibit kinase activity and expose the DHp domain to allow phosphatase activity of PhoR. These results are the first to point to a specific molecular mechanism for PhoU mediated modification of PhoR activity.

These studies have combined genetic, mutagenesis, computer modeling, and DCA analyses in studying the molecular interaction of PhoU and PhoR. It will be interesting to apply these techniques to identify residues involved in other signaling pathways and develop interaction models. For example, PhoU interaction with PstB appears to be weaker than PhoU interaction with PhoR based on our BACTH (Figure 6A and 6B), making it more difficult to directly identify residues essential for interaction. Using modeling and DCA, potential interacting residues may be identified. Efforts to isolate a complete signaling complex of PhoR, PhoU, and PstSCAB have been unsuccessful in the past. However, if the sites of interaction were identified, perhaps an entire signaling complex could be isolated and characterized using methods directed toward the specific interaction sites.

## Competing interests

The authors declare that they have no competing interests.

## Authors' contributions

SGG planned, performed experiments, prepared, and edited the manuscript. JM performed DCA, helped with analysis of data, and aided in preparing the manuscript. TD, TR, and ME helped in the scanning mutagenesis. PR helped with bioinformatic experimental planning and analysis. WRM designed experiments, conducted experiments, aided in data analysis, and aided in preparing and editing the manuscript.

## Supplementary Material

Additional file 1 **Table S1**. Primers used in this study. Click here for file

## Declarations

The publication costs for this article were funded by the Department of Microbiology and Molecular Biology at Brigham Young University, the College of Life Sciences at Brigham Young University, and the Public Health Service grant R15GM96222 from the National Institute of General Medical Sciences.

This article has been published as part of *BMC Genetics *Volume 16 Supplement 2, 2015: Selected articles from The 11th Annual Biotechnology and Bioinformatics Symposium (BIOT-2014): Genetics. The full contents of the supplement are available online at http://www.biomedcentral.com/bmcgenet/supplements/16/S2.
