# Supplementary material for: Genetic analysis, structural modeling, and direct coupling analysis suggest a mechanism for phosphate signaling in Escherichia coli
Source: BMC Genet. 2015 Apr 23;16(Suppl 2):S2. doi: 10.1186/1471-2156-16-S2-S2 (PMC4423584; doi:10.1186/1471-2156-16-S2-S2)
Supplement: Additional file 1 — Table S1. Primers used in this study. [file 1471-2156-16-S2-S2-S1.docx]

**Additional file 1**

Table S1 Primers used in this study

| Primer name | Sequence 5’-3’ |
| --- | --- |
| pstSFor | GCATGATCGTCATATGAAAGTTATGCGTACCACCGTC |
| pstBRev | GCATGACGTGGTACCTCAACCGTAACGACCGGTGAT |
| A147E | GCTGGACGCGTTCGAGCGGATGGACATTG |
| A147E antisense | CAATGTCCATCCGCTCGAACGCGTCCAGC |
| A147K | CGTGCTGGACGCGTTCAAGCGGATGGACATTGAC |
| A147K antisense | GTCAATGTCCATCCGCTTGAACGCGTCCAGCACG |
| R148A | TGGACGCGTTCGCGGCGATGGACATTGACG |
| R148A antisense | CGTCAATGTCCATCGCCGCGAACGCGTCCA |
| R148E | CTGGACGCGTTCGCGGAGATGGACATTGACGA |
| R148E antisense | TCGTCAATGTCCATCTCCGCGAACGCGTCCAG |
| D150A | GTTCGCGCGGATGGCCATTGACGAAGCGG |
| D150A antisense | CCGCTTCGTCAATGGCCATCCGCGCGAAC |
| 109 SphI | CGCGGAGTCGCTGCCCGCATGCGTGGTGCTGACCACGG |
| 109 SphI antisense | CCGTGGTCAGCACCACGCATGCGGGCAGCGACTCCGCG |
| 111 SphI | AGTCGCTGCCCGACGCGGCATGCCTGACCACGGAAGAGGG |
| 111 SphI antisense | CCCTCTTCCGTGGTCAGGCATGCCGCGTCGGGCAGCGACT |
| 113 SphI | GCCCGACGCGGTGGTGGCATGCACGGAAGAGGGCGGTA |
| 113 SphI antisense | TACCGCCCTCTTCCGTGCATGCCACCACCGCGTCGGGC |
| 115 SphI | CGACGCGGTGGTGCTGACCGCATGCGAGGGCGGTATTTTCTGGT |
| 115 SphI antisense | ACCAGAAAATACCGCCCTCGCATGCGGTCAGCACCACCGCGTCG |
| 117 SphI | CGGTGGTGCTGACCACGGAAGCATGCGGTATTTTCTGGT |
| 117 SphI antisense | ACCAGAAAATACCGCATGCTTCCGTGGTCAGCACCACCG |
| 119 SphI | GCTGACCACGGAAGAGGGCGCATGCTTCTGGTGTAACGGTCTGG |
| 119 SphI antisense | CCAGACCGTTACACCAGAAGCATGCGCCCTCTTCCGTGGTCAGC |
| 121 SphI | CCACGGAAGAGGGCGGTATTGCATGCTGTAACGGTCTGGCGC |
| 121 SphI antisense | GCGCCAGACCGTTACAGCATGCAATACCGCCCTCTTCCGTGG |
| 123 SphI | CAAGAATTTGTTGCGCCAGACCGCATGCCCAGAAAATACCGCCCTCTTCCG |
| 123 SphI antisense | CGGAAGAGGGCGGTATTTTCTGGGCATGCGGTCTGGCGCAACAAATTCTTG |
| 125 SphI | CGCAAACCAAGAATTTGTTGCGCGCATGCGTTACACCAGAAAATACCGCCCTC |
| 125 SphI antisense | GAGGGCGGTATTTTCTGGTGTAACGCATGCGCGCAACAAATTCTTGGTTTGCG |
| 127 SphI | TATTTTCTGGTGTAACGGTCTGGCATGCCAAATTCTTGGTTTGCGCTGGC |
| 127 SphI antisense | GCCAGCGCAAACCAAGAATTTGGCATGCCAGACCGTTACACCAGAAAATA |
| 129 SphI | GTGTAACGGTCTGGCGCAAGCATGCCTTGGTTTGCGCTGGCCGG |
| 129 SphI antisense | CCGGCCAGCGCAAACCAAGGCATGCTTGCGCCAGACCGTTACAC |
| 131 SphI | GGTGTAACGGTCTGGCGCAACAAATTGCATGCTTGCGCTGGCCGGA |
| 131 SphI antisense | TCCGGCCAGCGCAAGCATGCAATTTGTTGCGCCAGACCGTTACACC |
| 133 SphI | GTAACGGTCTGGCGCAACAAATTCTTGGTGCATGCTGGCCGGAAGATAAC |
| 133 SphI antisense | GTTATCTTCCGGCCAGCATGCACCAAGAATTTGTTGCGCCAGACCGTTAC |
| 135 SphI | GGCGCAACAAATTCTTGGTTTGCGCGCATGCGAAGATAACGGGCAGAACATCCTTA |
| 135 SphI antisense | TAAGGATGTTCTGCCCGTTATCTTCGCATGCGCGCAAACCAAGAATTTGTTGCGCC |
| 137 SphI | ATTCTTGGTTTGCGCTGGCCGGCATGCAACGGGCAGAACATCCTTAAC |
| 137 SphI antisense | GTTAAGGATGTTCTGCCCGTTGCATGCCGGCCAGCGCAAACCAAGAAT |
| 139 SphI | CTTGGTTTGCGCTGGCCGGAAGATGCATGCCAGAACATCCTTAACCTACTG |
| 139 SphI antisense | cagtaggttaaggatgttctggcatgcatcttccggccagcgcaaaccaag |
| 141 SphI | cgctggccggaagataacggggcatgcatccttaacctactgcgttac |
| 141 SphI antisense | gtaacgcagtaggttaaggatgcatgccccgttatcttccggccagcg |
| 143 SphI | taaactccgggtaacgcagtaggttgcatgcgttctgcccgttatcttccggccag |
| 143 SphI antisense | ctggccggaagataacgggcagaacgcatgcaacctactgcgttacccggagttta |
| 145 SphI | gcgtaaactccgggtaacgcaggcatgcaaggatgttctgcccgttatcttccggc |
| 145 SphI antisense | gccggaagataacgggcagaacatccttgcatgcctgcgttacccggagtttacgc |
| 147 SphI | tgcgtaaactccgggtagcatgctaggttaaggatgttctgcccgttatcttcc |
| 147 SphI antisense | ggaagataacgggcagaacatccttaacctagcatgctacccggagtttacgca |
| 149 SphI | gggcagaacatccttaacctactgcgtgcatgcgagtttacgcaatatctgaaaacgcgt |
| 149 SphI antisense | acgcgttttcagatattgcgtaaactcgcatgcacgcagtaggttaaggatgttctgccc |
| 151 SphI | ccttaacctactgcgttacccggcatgcacgcaatatctgaaaacgcgtg |
| 151 SphI antisense | cacgcgttttcagatattgcgtgcatgccgggtaacgcagtaggttaagg |
| 153 SphI | gagaaaaatcacgcgttttcagatagcatgcaaactccgggtaacgcagtaggttaagg |
| 153 SphI antisense | ccttaacctactgcgttacccggagtttgcatgctatctgaaaacgcgtgatttttctc |
| 155 SphI | gcgggcgagaaaaatcacgcgttttgcatgcttgcgtaaactccgggtaacgcagt |
| 155 SphI antisense | actgcgttacccggagtttacgcaagcatgcaaaacgcgtgatttttctcgcccgc |
| 157 SphI | cccggagtttacgcaatatctggcatgccgtgatttttctcgcccgctca |
| 157 SphI antisense | tgagcgggcgagaaaaatcacggcatgccagatattgcgtaaactccggg |
| 159 SphI | ccggagtttacgcaatatctgaaaacggcatgcttttctcgcccgctcaatctggtg |
| 159 SphI antisense | caccagattgagcgggcgagaaaagcatgccgttttcagatattgcgtaaactccgg |
| 181 SphI | gcatctggaaattcgcgtcatggcatgcacccacaaacagttgctgatgg |
| 181 SphI antisense | ccatcagcaactgtttgtgggtgcatgccatgacgcgaatttccagatgc |
| 183 SphI | ggaaattcgcgtcatgccttatgcatgcaaacagttgctgatggtggcg |
| 183 SphI antisense | cgccaccatcagcaactgtttgcatgcataaggcatgacgcgaatttcc |
| 185 SphI | cgcgtcatgccttatacccacgcatgcttgctgatggtggcgcgtgat |
| 185 SphI antisense | atcacgcgccaccatcagcaagcatgcgtgggtataaggcatgacgcg |
| 187 SphI | cgtcatgccttatacccacaaacaggcatgcatggtggcgcgtgatgtcacgcaat |
| 187 SphI antisense | attgcgtgacatcacgcgccaccatgcatgcctgtttgtgggtataaggcatgacg |
| 189 SphI | tgccttatacccacaaacagttgctggcatgcgcgcgtgatgtcacgcaataaggtac |
| 189 SphI antisense | gtaccttattgcgtgacatcacgcgcgcatgccagcaactgtttgtgggtataaggca |
| 191 SphI | gtaccttattgcgtgacatcgcatgccaccatcagcaactgtttgt |
| 191 SphI antisense | acaaacagttgctgatggtggcatgcgatgtcacgcaataaggtac |
| 193 SphI | cggtaccttattgcgtgcatgcacgcgccaccatcagcaactgttt |
| 193 SphI antisense | aaacagttgctgatggtggcgcgtgcatgcacgcaataaggtaccg |
| 195 SphI | tgaattcgagctcggtaccttagcatgcgacatcacgcgccaccatcagc |
| 195 SphI antisense | gctgatggtggcgcgtgatgtcgcatgctaaggtaccgagctcgaattca |

Table S1 Primers used in this study. This table lists the DNA primers used for cloning and mutagenesis used in this study. All sequences are listed 5’ to 3’.
